# Clinical Descriptive Study of Masturbatory Behavior Among Infants and Preschool Children: A Recent Observation From Iraq

**DOI:** 10.7759/cureus.11819

**Published:** 2020-12-01

**Authors:** Samer Dhaher, Khalifa Sharquie, Khalil Al Hamdi, Adil Noaimi

**Affiliations:** 1 Dermatology, University of Basrah College of Medicine, Basrah, IRQ; 2 Department of Dermatology and Venereology, College of Medicine, Baghdad University, Baghdad, IRQ

**Keywords:** masturbatory behavior, infants, preschool children, iraq

## Abstract

Background: Pleasurable feelings are normal human behaviors experienced not only by adults but even in infancy and childhood. These feelings might become habitual behavior in form of masturbation.

Objective: To evaluate the clinical criteria of infants and preschool children with masturbation habits who were described by parents as having unremitted genital pruritus.

Patients and methods: This is a case-series study describing cases of children with masturbation referred to the Department of Dermatology at Baghdad Medical City and Basra Teaching Hospital, central and southern Iraq, for four years (2014-2018). Children and their parents were carefully interrogated including detailed information about the problem. Local and general examinations were performed. Children with obvious genital lesions and those who proved to have urinary or gastrointestinal problems were not included.

Results: Forty-four children with masturbation were enrolled in the study (22 females and 22 males). The only reason for referral was unremitted genital pruritus. The mean age was 3.6 years. The frequency of masturbation events was variable; the mean length of events was 5 minutes. In 80% of children, masturbation happened at any time. Behavior during the event was prone or supine posturing with rubbing of the genital area with either hands or furniture followed by facial congestion, sweating, and sleep. The majority of children (68%) belonged to low social class families.

Conclusion: Masturbatory behavior is not uncommon in infants and preschool children and may mimic episodes of ongoing genital pruritus.

## Introduction

Masturbation or self-stimulation of genitalia is a common human behavior. It has been reported to occur in 90-95% of males and 50-60% of females during their life [1]. It is generally accepted that masturbation is a part of normal human sexual behavior [2] and is practiced in all ages and observed even in utero [3]. Masturbation during infancy and early childhood are often difficult to recognize because it may not involve the ordinary manual maneuver stimulation of genitalia, but instead, the stimulation is performed by different mechanical behavioral patterns, leading to great embarrassments to their parents [4]. It may be mistaken for other medical troubles like epilepsy [5], abdominal pain [6], and paroxysmal dystonia [7]. Thus, unnecessary investigations and even treatments might be used to manage these children [2,8], which will lead to anxiety and confusion for their parents. It has been noticed that childhood masturbation is often unrecognized neither by the parents nor by the physicians, especially when the genital manipulation is absent or when unusual posturing and movement occurred during the activity [9-11]. On reviewing the literature, we found only a few published isolated reports on gratification disorder (masturbation) during infancy and childhood. It seems that clinical awareness of this problem among physicians is lacking, leading to misdiagnosis and unnecessary treatments. Over the last few years, cases including infants and preschool children were referred to our departments for re-evaluation and assessment because of "itchy genitalia" that was totally unresponsive to any conventional treatment and all relevant laboratory tests were within the normal range. Even more, the parents were highly concerned and socially embarrassed because of their children's behavior. The diagnosis of masturbation was made based on characteristic points in the history and careful medical examination. The purpose of the present work is to perform a clinical evaluation and to describe the distinctive characteristics of this medico-social problem in infants and preschool children in the Iraqi population. 

## Materials and methods

This is a case series multicentric study carried out during the period from January 2013 to January 2018 at the Department of Dermatology and Venereology of Baghdad Teaching Hospital-Medical City and Basra Teaching Hospital, central and southern Iraq.

Children below the age of five years suspected to have masturbatory behavior were enrolled for an initial assessment. They were brought by their parents seeking treatment for "unremitted pruritus" of an organic cause in their children's genital area that could not be controlled by any medical therapies or because of a weird behavior of their children that causes social embarrassment to their parents.

During the interview, a careful, detailed history was obtained from children's parents regarding the age, sex, residency, duration, frequency and length of events, and time of occurrence. We inquired about any signs that appear on the child during or after the event and any tics or abnormal movements. A family history of psychological or psychiatric diseases was assessed. Previous diagnoses and therapies were also evaluated.

A careful local and general examination was performed with a special focus on the genital and perineal area. For children who were not previously investigated, a panel of tests was ordered, including blood counts, random blood sugar, blood urea and creatinine, urine and stool analysis, thyroid function, and liver function tests.

The main inclusion criteria were children aged five years or less, with a family complaint of stubborn "genital pruritus" of their siblings, no local genital or generalized skin pathology or systemic diseases detected on clinical examination, and all laboratory tests within normal referral values. Exclusion criteria included children with obvious localized or generalized skin lesions and yielded abnormal results of laboratory analysis.

The social background of families was estimated according to the Kuppuswamy socioeconomic status (SES) scale [12]. This scale had three components:

(1) Education level: ranges between seven points, as a maximum, for professionals and one point for the illiterate

(2) Occupation: ranged from 10 points for professional to one point for unemployed.

(3) Family income per month: This part was modified to be more relevant for the Iraqi community by simply changing the currency to Iraqi dinars and adding three zeros in front of the corresponding family income within the scale. The total sum for each patient was calculated and classified into five classes; class I: upper (26-29 points), class II: upper-middle (16-25), class III: lower-middle (11-15), class IV: upper-lower (5-10), and class V: lower class (<5 points). Data were collected and analyzed using the Statistical Package for the Social Sciences (SPSS), version 22 (IBM Corp., Armonk, NY). For categorical data, we use frequencies and percentages and for continuous data, we use means ± standard deviations.

The study was approved by The Ethical Committee of College of Medicine, Basra University (Approval No.: 005-2016). The study protocol was carefully explained to the parents of all participants and informed written consent was obtained from them.

## Results

Forty-four children were seen to have masturbation: 22 (50%) females and 22 (50%) males. Twenty-five (56.8%) cases were registered in Baghdad and 19 (42.2%) in Basra. Their demographic features are shown in Table 1. No family history of psychological or psychiatric diseases was found.

**Table 1:** Demographic characteristics of studied participants (No. = 44)

Parameter	Result
Sex	
Male	22 (50%)
Female	22 (50%)
Mean age of presentation ± SD (range)	3.2 ± 0.41 years (1.5-5 years)
Mean age of onset ± SD (range)	3.2 ± 0.8 years (11 months-4.5 years)
Mean duration ± SD (range)	2 ± 2.6 months (1-6 months)
Residency	
Rural	12 (27.2%)
Urban	32 (72.8%)
Initial diagnosis	
Unidentified cause	20 (45.5%)
Neurodermatitis	14 (31.8%)
Urinary tract infection	6 (13.6%)
Diaper dermatitis	4 (9%)
Socio-economic level	No. (%)
Class I (upper)	3 (6.8)
Class II (upper-middle)	3 (6.8)
Class III (lower-middle)	8 (18.1)
Class IV (upper-lower)	20 (45.4)
Class V (lower)	10 (22.7)

The main reasons for referral are summarized in Table 2.

**Table 2:** The main reasons for referral with the number and percentage of children with masturbation to a tertiary hospital

Reasons for referral	Number of cases	Percentage
1- Unidentified organic cause	9	20.5
2- Social embarrassment	5	11.3
3- Both	30	68.2

When we asked the parents to describe this problem in more details, they told the examining doctors that the event occurred in episodes that ranged from one to 10 times per day (mean: 4±2), and most of the time, the children do it in front of family members or guests, which causes great embarrassments.

Two masturbatory behaviors were described. In the first, the episode begins when the child laid down in a prone position putting their genitalia on a hard object like the edge of the furniture or bed mattress or pillow; without using their hands, they started to move in a repetitive back and forth rubbing their genitalia on the mattress for few minutes, and after that, the movement stops suddenly with congestion in the face and light sweating. The majority of them went to sleep. No ejaculation or passing fluid was observed from their genitalia.

In the second type of behavior, the child lay on his or her back and then start manual scratching the genital area for several minutes despite the parents' attempt to keep their hands away from the area. The parents did not report any habitual tics like thumb sucking or abnormal movements of their kids during or between the episodes.

The episode happened at any time in 35 (80%) children and only nocturnal in nine (20%). Analysis of socioeconomic background revealed that the majority of the affected children had originated from a low socio-economic class level. 

## Discussion

Pleasurable feelings and gratification are a well-known sensation that is experienced by infants and children when their genitalia are manipulated by their mother during napkin changes. This fact has been recognized and well reported in infants. However, gratification might become more evident and exaggerated in some children. Many of them learn this experience well, and they try to do it repeatedly to get some pleasure. If this sensation is repeated frequently, it will become a habit in the form of masturbation. Because they have an immature reproductive system and sexuality, masturbation in this age group is without ejaculation or passing fluid from their genitalia.

Masturbatory behavior is not rare among infants and preschool children. Most of the time, it is easily overlooked by the examining doctors and may cause confusion or misdiagnosis and anxiety for their parents. The parents often consult their doctors because of the social embarrassment caused by this "odd" behavior of their children. Parents often believe that a hidden and not yet diagnosed skin problem in the genital area triggers such unremitting pruritus. Unlike western communities, family concern and embarrassment about sex issues are significant in our conservative culture; therefore, parents of a child masturbating overtly in non-private places become concerned, and surely, ask a doctor for help. This finding was similarly noticed in other studies [13]. Most of the published studies about childhood masturbation were originally conducted in either psychiatric or pediatric clinics. In this study, we attempted to report on childhood masturbation being evaluated only from a dermatological point of view because the diagnosis was largely dependent on the parent's tale and observation, who describes persistent episodes of genital itching of their kids. Therefore, a small number of cases in this study may be attributed to patient selectivity and the inclusion criteria.

While previous studies have shown the higher prevalence of this behavior in females than males [3], no gender predilection was found in our study, this can be explained by the fact that both sexes are commonly practicing this habit [14], and the parental concern and worry about their children do not discriminate between the two genders.

In our observation, although no other abnormal behavior, tic, or habit was noticed, the psychological profile of children yet to be determined, but the parents, for social reasons, were reluctant to accept the idea of a psychological background for this behavior, especially since most of these children were under three years old. As the psychological aspect of childhood masturbation has been addressed in many of its aspects, the scientist Freud in his psychoanalytic theory, pointed out that masturbation is the main content of infantile sexuality and it was a universal occurrence during infancy as an autoerotic behavior for the purpose of sexual satisfaction and no longer be considered pathological [15]. However, a recent study showed that masturbation might be more associated with psychological disorders [16,17].

Although the higher rate of sexual behavior in children is directly related to the level of education and social level [18,19], most of the studied children originated from low socio-economic level, where emotional deprivation in these societies could be compensated by performing masturbation. To our knowledge, this report is the first observation among Iraqi children perhaps the reasons for the increase in this behavior in recent years are due to the compelling circumstances that Iraqi society has gone through, including disasters and conflicts [20].

Management of these cases is often by the reassurance of their parents as there is no specific skin or genital problem to be worried about; besides, this problem is often self-limiting and might disappear over time. Nevertheless, distracting the child by allowing him/her to engage in diverting activities during the episode with low doses of sedative might be prescribed for some of these children. 

## Conclusions

In conclusion, masturbatory behavior among preschool children is a real problem, often causing confusion and misdiagnosis. It should be considered in any child with persistent genital pruritus with no aberrant cause. Medical education among doctors and the public is required to avoid the consequence of misdiagnosis of such benign medical conditions.
